# The carbon price: a toothless tool for material efficiency?

**DOI:** 10.1098/rsta.2016.0374

**Published:** 2017-05-01

**Authors:** Alexandra C. H. Skelton, Julian M. Allwood

**Affiliations:** Department of Engineering, University of Cambridge, Cambridge, UK

**Keywords:** material efficiency, cost pass-through, steel

## Abstract

This article explores whether a carbon price will effectively encourage the more efficient use of greenhouse gas intensive materials such as steel. The article identifies a range of distortions that arise when some of the restrictive assumptions of neoclassical economics are relaxed. Distortions occur due to the sequential nature of decision-making along supply chains, due to imperfect competition and due to government intervention to reduce the risk of carbon leakage. If upstream sectors do not pass on carbon costs, downstream sectors do not have the opportunity to react. Of the distortions identified, compensation mechanisms that reduce the risk of carbon leakage are likely to act as the greatest hinderance to appropriate incentives for the more efficient use of steel in the UK: as things currently stand, unless upstream companies are encouraged to make windfall profits, incentives downstream are weakened. The article concludes by exploring policy options to address the distortions identified, including efforts to reinstate the carbon price downstream and efforts to remove other distortive taxes.

This article is part of the themed issue ‘Material demand reduction’.

## 1. Introduction

Greenhouse gas (GhG) emissions are a textbook example of an externality. Activities undertaken by businesses, households and governments that emit GhG emissions impose a cost on society that, in the absence of government intervention, is not reflected in the costs faced by relevant decision-makers. To address this market failure, economists have sought to identify the most appropriate policy intervention to ensure that these societal costs are adequately taken into account, and that consequent GhG emissions abatement effort is allocated efficiently across all available options. As explained by Aidt *et al.* [1] in this theme issue, the efficient allocation of abatement effort should ensure both ‘production efficiency’ (ensuring that a maximum possible output is achieved from a given set of inputs such as capital, labour and energy) and ‘product mix efficiency’ (ensuring that the goods and services that are made reflect consumer preferences for these goods and services), taking into account the cost to society of GhGs emitted in the course of production.

In theory, a carbon price—levied either as a tax or via a cap-and-trade scheme—achieves these criteria by pricing GhG emissions at source, causing a chain of price changes along supply chains that reflect the social cost of GhG emissions embodied in intermediary, and subsequently final, goods. These price changes should then offer appropriate incentives for both upstream GhG abatement activity (such as the pursuit of renewable energy generation) and downstream GhG abatement activity (including the pursuit of greater energy efficiency in industry, the pursuit of greater efficiency in the use of embodied emissions-intensive materials, and the substitution of demand towards less emissions-intensive options), restoring ‘production efficiency’ and ‘product mix efficiency’ at the new set of prices.

Aidt *et al.* [1] stress that a key advantage of carbon prices over other forms of intervention is the flexibility that carbon prices offer over how GhG emissions abatement is achieved: whether through upstream, supply-side options, or downstream, demand-side options. Given the scale of the ambition to limit global temperature increases to less than 2^°^C set out in the Paris Agreement, encouraging the full gamut of GhG emissions abatement options becomes not just desirable (in an effort to meet the target efficiently), but necessary (if the target is to be met at all) because, taking into account the cumulative emissions already released into the atmosphere, the necessary reduction in emissions cannot be met realistically through supply-side emission reductions alone [2].

Despite the theoretical advantages of carbon prices, there are many difficulties associated with implementing this instrument under real world, ‘second best’ conditions. These include: that power structures and diverse interests foster a fragmented global institutional ‘regime complex’ to manage climate change, that does not lend itself well to the global implementation of a single instrument [3]; that subsequent unilateral action to enforce a carbon price risks the relocation of GhG emissions intensive production, referred to as ‘carbon leakage’ [4,5]; and, that the burden of carbon prices falls disproportionately on low-income households [6], threatening the social stability of fully internalizing the social cost of GhGs (as discussed by Kallis [7]).

In the absence of a single global carbon pricing scheme, a number of localized efforts to price carbon have been put into place. In a recent study for the World Bank, Ecofys [8] identifies 38 distinct carbon pricing schemes globally that cover approximately 12% of global GhG emissions. These include regional schemes (e.g. the European Union Emissions Trading Scheme (EUETS)), national schemes (e.g. the Swedish carbon tax), sub-national schemes (e.g. the emissions trading pilot scheme in China’s Guangdong province) and city-level schemes (e.g. Quebec’s carbon tax). Owing to the difficulties identified above, the imposed cost of carbon tends to be low: 85% of GhG emissions that fall under the identified schemes are priced at less than $10/tCO_2_ [8]. This is far below the social cost of $220/tCO_2_ in 2015 estimated by Moore & Diaz [9] and also below the social cost of carbon estimated by the US Environmental Protection Agency ranging from $11/tCO_2_ to $105/tCO_2_ in 2015 across different scenarios [10]. Clearly, to date, it has not been possible to implement carbon prices in practice as they are envisaged in theory.

Recognizing the importance of encouraging the full gamut of GhG emission reduction options, and the compromises that have to be made in implementing carbon prices in real-world circumstances, the purpose of this article is to question whether carbon prices will effectively encourage downstream emissions abatement. The focus is on steel, because: (i) steel is an embodied emissions-intensive bulk material for which there are no lower GhG emissions-intensive scalable substitutes [11]; (ii) there are abundant technical opportunities to improve the efficiency with which steel is used (as identified for example in [12], which shows that up to 50% of steel in office blocks is surplus to requirements, and explored extensively in [13]); and (iii) improving downstream material efficiency is vital if the sector is to bear its share of GhG emission reduction targets [14].

This article does not go further into the more general issues with implementing carbon prices touched upon above that reduce the likelihood that carbon prices will be implemented, thus limiting incentives for abatement across the board. Instead, this article focuses specifically on reasons why incentives downstream may be weaker than incentives upstream, i.e. reasons why an abatement option with a particular cost may be more likely to be implemented if it applies upstream (e.g. installing renewable energy options) rather than downstream (e.g. exploiting opportunities to improve yield in the automotive sector). Despite the provocative title of this article, the intention is not to suggest that carbon pricing schemes should not be pursued, but instead to encourage the policy community to be aware of potential distortions to downstream incentives offered by carbon prices and to act to try to reduce such distortions. The next section (§2) provides a brief overview of the incentives offered by a carbon price within the traditional, neoclassical economic framework. Subsequent sections (within §3) then explore how distortions to downstream incentives arise when various neoclassical assumptions are relaxed.

## 2. The incentives offered by a carbon price

Within the standard economic model, firms seek to maximize profits for a given set of input prices. Following Varian [15], p. 580, the profit maximization problem for a firm in the steel sector that is faced with a carbon price is
2.1
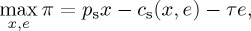
where *π* represents the firm’s profits, *p*_s_ is the price of steel, *x* is the chosen level of output of the firm, *e* is the GhG emissions associated with output *x*, *c*_s_(*x*,*e*) is the continuous cost function that describes the cost of producing output *x* with associated emissions *e*, and *τ* is the carbon tax (£/tCO_2_) levied on GhG emissions caused by producing the firm’s output. The first-order conditions for solving this optimization problem are
2.2
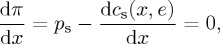

2.3
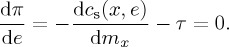


Rearranging these first-order conditions gives two decision rules for the firm: (i) choose a level of output such that the marginal cost of producing the final unit is equal to the marginal revenue received from selling that final unit (equation (2.4)); and (ii) choose to reduce emissions until the marginal cost of abatement is equal to the tax (equation (2.5)),
2.4
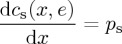
and
2.5
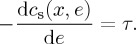


**Figure 1.**
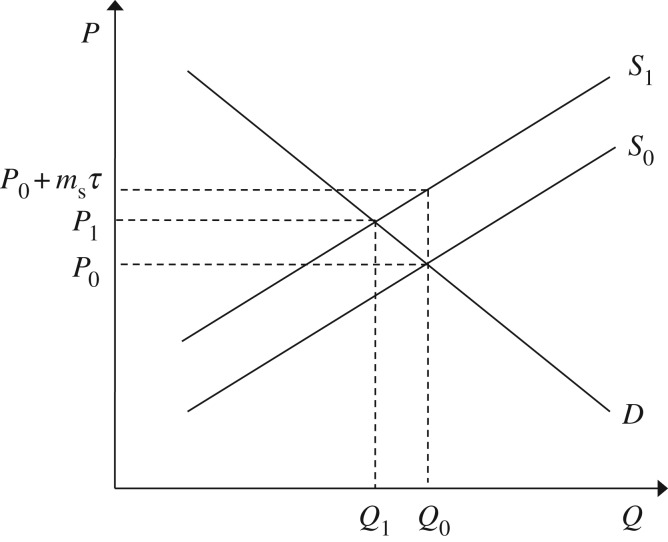
Steel market equilibrium following the introduction of a carbon tax.

Figure 1 shows the effect of these firm-level decisions on the steel market. The market starts in the pre-tax equilibrium (*Q*_0_,*P*_0_) where supply (*S*_0_) equals demand (*D*). Following the imposition of the carbon price *τ*, firms within the steel sector choose to abate GhG emissions until the cost of abatement is equal to the tax (in accordance with equation (2.5)), causing an increase in the cost of production that is equal to the embodied emissions within the product prior to any abatement activity (*m*_s_) multiplied by the carbon tax (*τ*). This increase in cost causes the supply curve to shift from *S*_0_ to *S*_1_. The resulting increase in price, from *P*_0_ to *P*_0_+*m*_s_*τ*, means that supply exceeds demand at the original level of output *Q*_0_. Consumers of steel see the increase in price and adjust their demand accordingly (e.g. by pursuing material efficiency measures), reducing demand from *Q*_0_ to *Q*_1_.

In figure 1, the carbon price provides appropriate incentives for both upstream GhG emissions abatement and downstream GhG emissions abatement: firms within the steel sector and consumers of steel both face an increase in the unit cost of steel that is determined by the GhG emission intensity of steel-making, priced according to the carbon price (*m*_s_*τ*). If the carbon price is levied through a cap-and-trade scheme the market price will automatically adjust in order to ensure that the desired aggregate level of abatement (the cap) is met. If, instead, the carbon price is administered as a tax, the government must actively manipulate the level of the tax following the observed response in order to ensure that the desired target is met.

## 3. Issues with using a carbon price to motivate material efficiency

The purpose of this section is to explore a range of arguments that may call into question the common assertion that a carbon price will effectively encourage downstream emissions abatement as described above. The section covers the sequential nature of decision-making along supply chains, the possibility of imperfect cost pass-through and the implications of carbon leakage and associated compensation measures on downstream GhG abatement decisions. The distortions to downstream incentives identified in this section arise from relaxing the restrictive assumptions of the traditional neoclassical model: the assumption that the economy is in equilibrium (in §3a); the assumptions that firms operate under perfect competition and seek only to maximize profits (in §3b); and the assumption that governments can implement optimal policies (in §3c).

### (a) Sequential decision-making

Firms within supply chains do not see the cost of a carbon price simultaneously. Instead the tax is levied at the point at which emissions occur, and then passed on in the form of higher intermediary and subsequently final product prices. Following the introduction of a carbon tax, the change in costs seen by downstream firms depends on the abatement decisions made by upstream firms. As shown in equation (2.5) in §2, in equilibrium, once upstream firms have duly abated in response to the imposed tax, the upstream abatement cost will equal the tax and downstream GhG emissions abatement will be duly incentivized; however, the tendency in neoclassical economics to focus on equilibrium conditions may mask problems that occur in the transition from one equilibrium (e.g. the pre-tax market equilibrium *Q*_0_*P*_0_ in figure 1) to another (e.g. the post-tax market equilibrium *Q*_1_*P*_1_ in figure 1).

To examine whether this transition between equilibria may be problematic in encouraging downstream emissions abatement, this section explores the incentives for abatement within a highly stylized supply chain. The supply chain, described in table 1, consists of three sectors: the energy sector, the steel sector and the construction sector. In order to focus specifically on the incentives for downstream GhG emissions abatement, only GhG emissions associated with the energy sector are taken into account. This stylized supply chain could represent the use of electricity (causing emissions from the energy sector) to melt scrap steel in electric arc furnaces (EAFs) (causing minimal direct emissions in the steel sector that are not taken into account here), in order to supply steel beams to the construction sector. Emissions associated with other inputs and with the use phase are not taken into account in this stylized model, and the supply chain is assumed to be linear (excluding feedback loops such as the energy sector’s demand for steel to build power stations).

**Table 1.** Stylized supply chain characteristics; where *a*_*i*,*j*_ represents the physical amount of output from sector *j* required to make one unit output from sector *i*; *m*_*i*_ represents the GhG emissions intensity per unit output in sector *i*; *n*_*i*_ represents the indirect GhG emissions released in other sectors in order to make one unit of output from sector *i*; *c*_*i*_ represents the cost of implementing the sector-specific abatement strategy in sector *i* (reduced carbon intensity, energy efficiency or material efficiency); and *α*_*i*_ is the cost of abatement in sector *i*, calculated by translating sector-specific abatement strategy costs into common £/tCO_2_ units. The sectors are denoted as follows: energy (e); steel (s); construction (c); household (hh).

	energy	steel	construction
demand (units)	*a*_c,s_⋅*a*_s,e_ (MJ/building)	*a*_c,s_ (t/building)	*a*_hh,c_=1 (buildings)
direct emissions (units)	*m*_e_ (tCO_2_/MJ)	*m*_s_=0 (tCO_2_/tsteel)	*m*_c_=0 (tCO_2_/building)
indirect emissions (units)	*n*_e_=0 (tCO_2_/MJ)	*n*_s_=*m*_e_⋅*a*_s,e_ (tCO_2_/tsteel)	*n*_c_=*m*_e_⋅*a*_s,e_⋅*a*_c,s_ (tCO_2_/building)
strategy cost (units)	*c*_e_ (£/tCO_2_)	*c*_s_ (£/MJ)	*c*_c_ (£/tsteel)
abatement cost equivalent (units)	*α*_e_=*c*_e_ (£/tCO_2_)	*α*_s_=*c*_s_/*m*_e_ (£/tCO_2_)	*α*_c_=(*c*_c_/*a*_s,e_)/*m*_e_ (£/tCO_2_)

Whereas, in §2, a firm’s cost of abatement was characterized by a continuous cost function (that rises to the level of the tax, as lower cost abatement opportunities are exhausted over time), here, a snapshot in time is taken to explore how appropriate incentives are in the transition from the pre-tax equilibrium (*Q*_0_*P*_0_ in figure 1) to the post-tax equilibrium (*Q*_1_*P*_1_ in figure 1). At this snapshot in time, firms in each sector face a fixed marginal cost of abatement (equal to *α*_e_ in the energy sector, *α*_s_ in the steel sector and *α*_c_ in the construction sector) that represents the cost in each sector of abating one additional unit of GhG emissions.

This abatement cost is not evident to firms in all sectors. Instead, firms are aware of the cost of the particular strategy that is available to them to reduce the GhG emissions generated in the energy sector through their activities: the energy sector can directly reduce GhG emissions per unit energy (for example by switching to renewable energy sources at a strategy cost, *c*_e_, measured in £/tCO_2_ units); the steel sector can reduce the amount of energy required to make a unit of steel (for example by pre-heating scrap steel prior to recycling at a strategy cost, *c*_s_, measured in £/MJ units); and the construction sector can reduce the amount of steel required to make a building (for example by using design software to reduce the over-specification of steel in buildings at a strategy cost, *c*_c_, measured in £/tsteel units).

**Figure 2.**
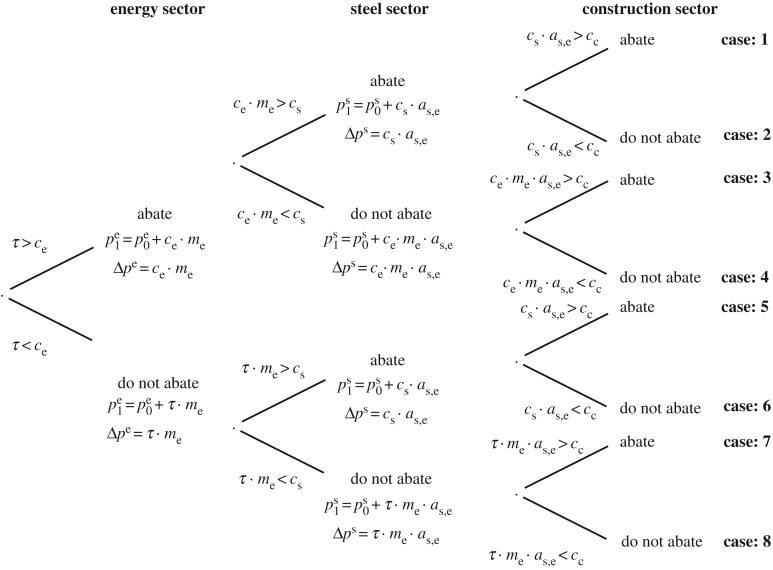
Decision tree showing sequential abatement decisions, where *τ* represents the carbon price; 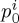 represents the price of one unit of output from sector *i* prior to introducing the carbon price; 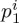 represents the price of one unit of output from sector *i* after introducing the carbon price; *c*_*i*_ represents the cost of implementing the sector-specific abatement strategy in sector *i* (reduced carbon intensity, energy efficiency or material efficiency) in sector-specific units as defined in table 1; *m*_*i*_ represents the GhG emissions intensity per unit output in sector *i*; and, *a*_*i*,*j*_ represents the physical amount of output from sector *j* required to make one unit output from sector *i*. The sectors are denoted as follows: energy (e), steel (s) and construction (c).

**Figure 3.**
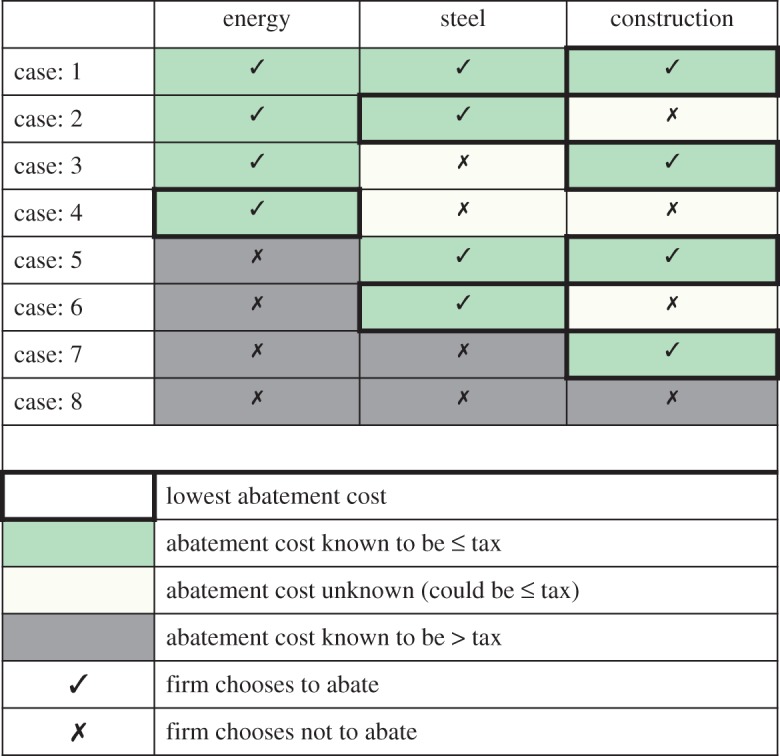
Summary of abatement decisions across different eventualities described in figure 2.

Following the logic of the optimization problem set out in §2, firms choose to implement each strategy if the marginal cost of the strategy is less than the marginal benefit, where the marginal benefit of implementing the strategy is not having to pay the increase in price of the relevant resource (i.e. the increase in the price of carbon for the energy sector, the increase in the price of energy for the steel sector and the increase in the price of steel for the construction sector). The decision tree in figure 2 captures all possible outcomes under the assumption that representative firms within each sector face a binary choice—to abate or not to abate—at the particular snapshot in time considered here. The relative cost conditions and resulting abatement decisions are summarized in figure 3 for each of the eight possible eventualities (referred to as cases and numbered according to figure 2). The full relative cost conditions in each case, including conversions between strategy costs (in mixed units outlined above) and abatement costs (in common £/tCO_2_ units), are provided in appendix A.

Figure 3 reveals that in all cases the cheapest abatement option is chosen. This means that, in accordance with economic theory, the sequential nature of decision-making does not violate the optimality condition that least-cost options are chosen first. If the cheapest option to abate emissions is to pursue material efficiency in the construction sector, then (under the assumptions made in this section) this option will be incentivized not just in the long-run post-tax equilibrium, but also in the short-run transition to that equilibrium.

Figure 3 also reveals a more modest potential bias against downstream options. In cases 2, 3, 4 and 6 described in figures 2 and 3, it is possible that viable abatement options (i.e. options that cost less than the carbon tax) in downstream sectors are overlooked. Across all of these eventualities upstream sectors have chosen to abate, meaning that downstream sectors do not face the full carbon tax (*τ*) but the upstream abatement cost (known to be less than *τ*). Consequently, it is possible that the downstream abatement cost lies between the upstream abatement cost and the tax (e.g. *α*_s_<*α*_c_<*τ*) and so that a viable abatement option is overlooked.

This means that whether or not an abatement option with a particular cost is chosen in the short run depends partly on the stage of the supply chain at which it is implemented: options that are viable (i.e. less costly than the carbon price) but sub-optimal (i.e. not the cheapest abatement option within the supply chain) are less likely to be chosen if they occur later in the supply chain. Over time, as abatement options that are applied earlier in the supply chain are exhausted, the cost of abatement in upstream sectors will rise to the level of the tax (in accordance with equation (2.5)) and all viable downstream abatement options will be incentivized. Nevertheless, should there be any increasing returns to scale in abatement activity (for example the opportunity to reduce costs by exploiting economies of scale or through learning-by-doing) upstream abatement activity would benefit first.

This section has sought to establish whether the sequential nature of decision-making along supply chains may distort incentives for downstream GhG emissions abatement through strategies such as material efficiency. The analysis has revealed that least-cost options will be incentivized regardless of whether they occur upstream or downstream. This is true both in the long-run equilibrium when the marginal cost of abatement is equal to the tax and in the short-run transition to that equilibrium. In addition, the analysis has revealed that, in the short run, viable but sub-optimal downstream abatement options (i.e. options that cost less than the tax but are not the cheapest option within the supply chain) may be overlooked.

### (b) Imperfect cost pass-through

Previous sections of this article assumed that firms fully pass on any increases in costs incurred: in figure 1, when the unit cost of steel-making increased by *m*_s_*τ*, the price of steel initially increased by the same amount, and similarly at each stage in the decision tree in figure 2, firms fully passed on any increases in costs (whether due to the upstream abatement cost or the cost of the tax) to their customers in the next tier of the supply chain. This section explores reasons for imperfect cost pass-through, i.e. reasons why companies may absorb costs rather than pass them on to their customers. The section covers imperfect competition (§3b(i)) and alternative objectives and accountancy practices (§3b(ii)), before providing a brief summary of findings (§3b(iii)).

This section draws on the literature on tax pass-through, a body of work that has been primarily focused on two areas: (i) the theory of tax incidence which is concerned with how the burden of taxes is shared between consumers and producers (e.g. [16]); and (ii) the analysis of the risk of carbon leakage, i.e. the risk that unilateral implementation of a carbon tax will cause substitution towards production activity outside the jurisdiction of that tax (e.g. [17]). There has been little investigation of the implications of imperfect cost pass-through for the incentives for downstream GhG emissions abatement (with [18] being the notable exception).

#### (i) Imperfect competition

The analysis so far has adopted the typical neoclassical assumption that firms operate under perfect competition. Within this theoretical construct, competition is so great that firms are unable to make supernormal profits (i.e. profits that would encourage more firms to enter the sector), and price is equal to marginal cost. It follows that any increases in costs, due to the imposition of a tax, translate directly into increases in prices seen by customers, as firms do not have the capacity to absorb costs by reducing profits. When the assumption of perfect competition is relaxed, firms do not take market prices as given, but instead can have some influence on prices. In this situation, firms can restrict output, raising prices and allowing supernormal profits to be made. Rather than being automatic (as in the case of imperfect competition), the rate of cost pass-through then becomes the outcome of a strategic decision made by a firm that has a choice to either pass on costs to customers (reducing demand) or absorb costs in profits.

Before exploring the strategic cost pass-through decision under imperfect competition, it is important to distinguish between two types of cost pass-through to help interpret findings with respect to the incentives for downstream GhG abatement through material efficiency. For the purposes of this article: the ‘initial cost pass-through rate’ describes the initial increase in price relative to the magnitude of the tax imposed (((*P*_0_+*m*_s_*τ*)−*P*_0_)/*m*_s_*τ*=100% in figure 1); and the ‘consequent cost pass-through rate’ describes the post-tax equilibrium price relative to the magnitude of the tax imposed ((*P*^1^−*P*^0^)/*m*_s_*τ* in figure 1). The perfectly competitive case can then be described as having an initial cost pass-through rate of 100% and a consequent cost pass-through rate (*ρ*_pc_) of (*P*^1^−*P*^0^)/*m*_s_*τ* in figure 1, shown by Weyl & Fabinger [16] to be determined by the ratio of the elasticity of demand (*ϵ*_D_) to that of supply (*ϵ*_S_) according to
3.1
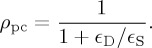


It follows that downstream incentives are distorted if the initial pass-through rate is imperfect (i.e. not equal to 100%), but that a consequent pass-through rate of less than 100% can be consistent with efficient downstream incentives, and arises because this measure takes into account the dampening effect on prices caused by the demand response (from *Q*_0_ to *Q*_1_ in figure 1) to the initial price increase.

**Figure 4.**
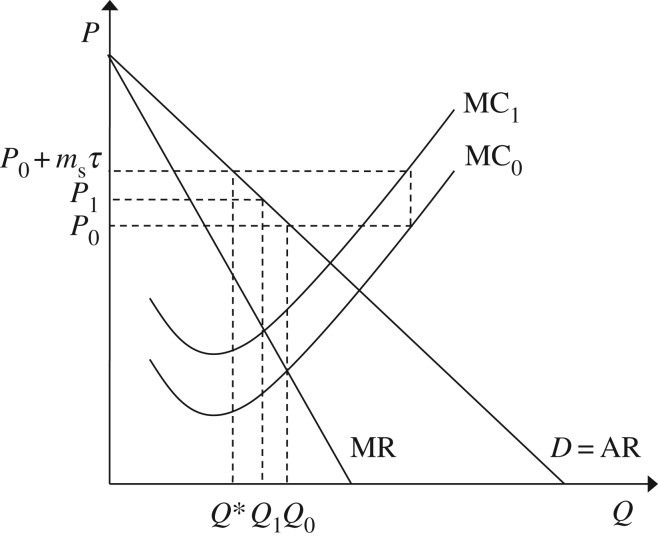
Carbon tax pass-through under monopoly.

To explore the incentives for material efficiency under imperfect competition, figure 4 shows the decision framework for the monopolist. Before the introduction of the tax, the monopolist chooses to restrict output to ensure that marginal cost (MC_0_) is equal to marginal revenue (MR) resulting in the pre-tax equilibrium (*Q*_0_,*P*_0_). The tax is introduced and shifts the monopolist’s marginal cost curve from *MC*_0_ to MC_1_. Again the monopolist makes the strategic decision to ensure that price is equal to marginal cost, resulting in the post-tax equilibrium (*Q*_1_,*P*_1_). The ratio of the resulting increase in price (*P*_1_−*P*_0_) to the initial tax per unit (*m*_s_*τ*), referred to here as the consequent cost pass-through rate (*ρ*_m_ under monopoly), is determined by equation (3.2) and is dependent on not only the ratio of the elasticity of demand (*ϵ*_D_) to that of supply (*ϵ*_S_) but also a measure of the curvature of demand (*ϵ*_ms_) according to Weyl & Fabinger [16],
3.2
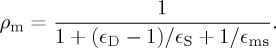

If the initial cost pass-through rate in figure 4 were perfect, i.e. if the full cost of the tax were passed on to consumers, the price to consumers would initially increase to *P*_0_+*m*_s_*τ*. Figure 4 shows that this price increase would be greater than the strategic optimal choice by the monopolist described in equation (3.2), and so would cause a greater demand response (with demand decreasing from *Q*_0_ to Q* rather than to *Q*_1_). It follows that monopolistic competition does distort downstream incentives: in the example shown in figure 4, the monopolist chooses not to initially pass on the full cost of the tax, resulting in a demand response (*Q*_1_) that is less than the response (Q*) that would be expected if unit prices increased to fully reflect the emissions intensity of production (*m*_s_*τ*). In this case, a portion of the tax on carbon is effectively acting as a tax on monopolistic profits.

It is important to remember that, under imperfect competition, demand is constrained and product prices are higher than the optimal level that would be achieved under perfect competition. This has implications for the level of downstream abatement that is incentivized before the tax is introduced. As prices are inflated under imperfect competition, the equilibrium demand prior to the introduction of the tax is lower than the perfectly competitive optimum. Therefore, although the effect of a carbon tax on downstream abatement activity may be constrained (if the strategic level of initial cost pass-through chosen under imperfect competition is less than or equal to 100%), the absolute level of abatement may be higher (if prices are kept strategically high under imperfect competition). The resulting interplay between the regulation of imperfect competition and the environment is considered by Requate, who states that ‘the regulator has to know the whole vertical structure, including the degree of market power at each step of the production chain, in order to determine the optimal tax rate, or the level of some other policy instrument’ [19], p.87. Simply relying on carbon prices to optimally distribute abatement effort does not work under imperfect competition.

#### (ii) Alternative objectives and accountancy practices

Aside from imperfect competition, a number of reasons for imperfect cost pass-through have been identified in the literature, including that firms may prioritize the pursuit of market share rather than profit (as discussed in [20]) and that firms may choose not to pass on the opportunity cost of freely obtained permits (as surveyed in [21], who found that 40% of companies surveyed classified freely allocated emission reduction permits as intangible assets with zero value). Both of these situations cause imperfect initial cost pass-through, distorting incentives for downstream material efficiency. If upstream companies do not initially pass on the full cost of the tax, downstream companies are not incentivized to react appropriately.

#### (iii) Summary: imperfect cost pass-through

This sub-section revealed a range of reasons for imperfect cost pass-through, including imperfect competition, alternative objectives of firms and imperfect accountancy practices. Each of these reasons could reduce the ‘initial cost pass-through rate’ and so distort downstream incentives.

### (c) Distortions caused by government intervention

So far, this article has focused on government intervention to impose a cost on GhG emissions that reflects the social cost of the environmental damage caused by GhG emissions. This section explores possible distortions to downstream incentives caused by other forms of government intervention, in particular: distortions caused by compensation schemes that seek to reduce the risk of carbon leakage due to unilaterally imposed carbon taxes (§3c(i)) and distortions caused by labour taxes that increase the price of labour relative to the price of materials (§3c(ii)), before briefly summarizing the findings in this section (§3c(iii)).

#### (i) Compensation measures

As explained in the Introduction, the realities of implementing a carbon price are challenging, and so far only local, national and regional emissions pricing mechanisms have been implemented. The failure to secure a consistent global carbon price poses the risk that unilaterally implemented carbon taxes will simply encourage production activity outside the jurisdiction of these schemes. In response, governments have put in place compensation schemes that aim to reduce the financial burden of carbon taxes on energy-intensive sectors. These compensation mechanisms may distort incentives downstream.

Within the UK, there are two carbon pricing instruments that affect the steel sector: the European Emissions Trading Scheme (EUETS) and the UK Carbon Price Support (UK CPS; a carbon floor price that was introduced in the UK in 2013 to supplement the EUETS price). The EUETS covers direct emissions from a group of sectors including the energy sector and steel sector and so applies to both primary steel-making (whereby steel is reduced from iron ore in a blast furnace and a basic oxygen furnace (BF-BOF), causing direct GhG emissions) and secondary steel-making (whereby scrap steel is remelted in an Electric Arc Furnace (EAF), causing indirect emissions upstream in the electricity sector) within the EU. The UK carbon price floor is a national intervention that applies to UK electricity generators only and so is only relevant for secondary steel-making on UK soil. The UK CPS sets a floor price for the EUETS, which is to say that when the EUETS price is below that of the UK CPS (as is currently the case) then the UK CPS is charged on emissions associated with UK electricity generation.

Owing to concerns over the adverse competitive impacts of the EUETS and UK CPS on downstream energy-intensive sectors (such as the steel sector) that are particularly susceptible to carbon leakage, mechanisms to reduce costs associated with these measures have been introduced. In line with EU guidelines, the UK government offers compensation for indirect carbon costs in sectors that face international competition outside the EU [22]. Companies within the steel sector are eligible for this relief if they spend more than 5% of their gross value added (GVA) on indirect carbon taxes (the combined costs of the EUETS and the UK carbon price floor). The formula for calculating the maximum potential level of compensation is
3.3
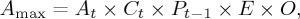
where *A*_*t*_ is the maximum permissible aid intensity (currently set at 85%), *C*_*t*_ is the assumed carbon emissions factor for UK electricity (set at 0.58 tCO_2_/MWh, based on the average carbon content of UK electricity supplied by fossil fuel plants), *P*_*t*_ is the carbon price (either the EUETS price currently at approximately £5/tCO_2_ or (if applicable) the UK carbon floor price at £18/tCO_2_), *E* is a product-specific electricity consumption efficiency benchmark set by the European Commission (0.49 MWh/*tsteel* for EAF steel) and *O* is the baseline output [22].

Aside from this compensation mechanism (which applies to both the EUETS and the UK carbon price floor), the burden of the EUETS on industry has been reduced by allocating a share of emission reduction permits for free. This year over 95% of the EUETS permits (EUAs) required by the UK steel industry were freely allocated to businesses in the sector. Indeed, the European Commission [17] states that ‘Allocation of EUAs [EUETS emissions permits] to the iron and steel sector has exceeded verified emissions continuously since the start of the EU ETS in 2005’.

Table 2 provides a summary of the GhG emission costs and accompanying compensation measures faced by primary and secondary steel producers in the UK. The table is based on estimates of the average GhG emissions intensity of primary (BF-BOF) and secondary (EAF) steel-making from Griffin *et al.* [23], making the simplifying assumption that all GhG emissions associated with primary steel-making and 15% of GhG emissions associated with secondary steel-making are direct; the remainder being indirect due to electricity consumption (based on [24]). Compensation rates are calculated under the assumption that 95% of allowances for direct emissions under the EUETS are allocated for free, that the maximum permissible aid intensity for indirect carbon cost compensation is 85% [22] and that secondary steel-makers are able to produce steel at the benchmark emissions intensity equal to 0.28 *tCO*_2_/tsteel (based on equation (3.3) 0.58 tCO_2_/MWh*0.49 MWh/*tsteel*). Table 2 provides an example of the compensation calculations based on credible estimates; actual compensation rates will vary from plant-to-plant depending on the emissions intensity of production activity relative to the benchmark.

**Table 2.** UK steel sector carbon prices and associated compensation schemes.

measure	units	BF-BOF	EAF
**emissions intensity**	**tCO**_**2**_/**t steel**	**2.3**	**0.6**
*of which direct*	*tCO*_*2*_/*t steel*	*2.3*	*0.1*
*of which indirect*	*tCO*_*2*_/*t steel*	*0*	*0.5*
**Carbon prices**			
EU ETS price: direct emissions	£/tCO_2_	5	5
UK CPS price: indirect emissions	£/tCO_2_	18	18
**carbon costs (excluding compensation)**	**£**/**t steel**	**11.4**	**9.6**
*of which EU ETS*	£/*t steel*	*11.4*	*0.5*
*of which UK CPS*	£/*t steel*	*0*	*9.2*
**carbon costs compensation**	**£**/**t steel**	**−10.8**	**−8.2**
*of which free allowances EU ETS*	£/*t steel*	*−10.8*	*−0.4*
*of which indirect carbon cost compensation UK*	£/*t steel*	*0*	*−7.8*
**carbon costs (net compensation)**	**£**/**t steel**	**0.6**	**1.4**
**effective carbon tax paid**	**£**/**tCO**_**2**_	**0.3**	**2.3**

**Figure 5.**
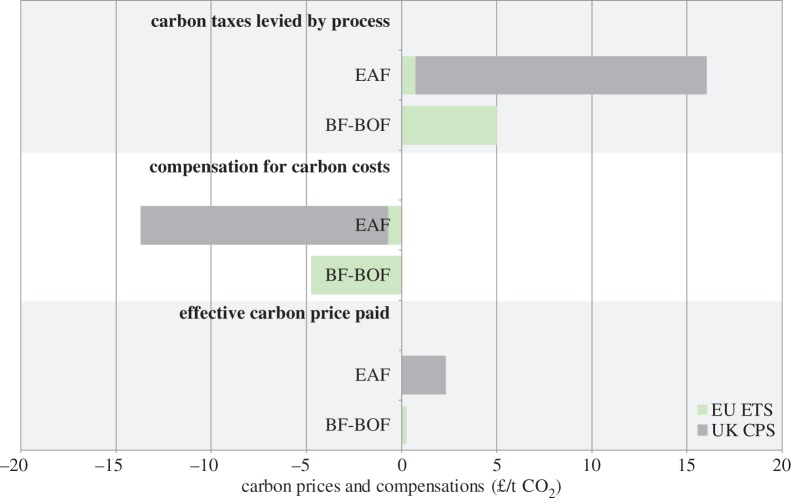
Compensation for carbon costs. (Online version in colour.)

Figure 5, based on table 2, demonstrates the perverse situation—caused by the limited sectoral coverage of the UK CPS, the difference in the carbon prices charged across the schemes and differing rates of compensation—that primary steel-makers are effectively taxed at a carbon price that is 10% of that levied on secondary steel-makers. This encourages primary steel-making over secondary steel-making despite the emissions savings associated with secondary steel-making (which accounts for a quarter of the emissions per tonne of steel associated with primary steel-making). More importantly for this article, the table shows that expenditure on carbon taxes (that are levied at rates far below estimates of the social cost of carbon) is further reduced radically through compensation schemes: primary steel-makers are effectively charges 30p/tCO_2_ and secondary steel-makers are effectively charged £2.30/tCO_2_ despite the fact that the EUETS charges approximated £5/tCO_2_ and the UKCPS is set at £18/tCO_2_. The potential distortions caused by these compensation mechanisms to downstream incentives are twofold. Firstly, if companies do not pass on the opportunity cost associated with freely allocated EUETS allowances, the downstream incentives offered by this scheme will be severely limited. Secondly, the method by which energy-intensive industries are currently compensated for their carbon costs (in line with equation (3.3)) means that they do not see the full cost of carbon embodied in their production decisions so are barely incentivized to reduce their use of embodied emissions inputs. Although steel-makers may be expected to pass on the opportunity cost of freely obtained allowances, they would not be expected to pass on the cost of indirect carbon costs for which they are compensated.

#### (ii) Distortions caused by labour taxes

So far, this article has focused on carbon prices and associated compensation schemes; however, other forms of government intervention can have unwanted side-effects on the incentives for material efficiency. Governments impose taxes on labour in order to redistribute income from high-income households to low-income households, and in order to raise revenues for the provision of public services. This tax can have unwanted side-effects as it increases the cost of labour, encouraging substitution towards other factors of production and increasing unemployment. Many downstream material efficiency measures necessitate the substitution of labour for embodied emissions-intensive materials. For example, improving communication between fabricators and designers (i.e. increasing design time) in both the automotive sector and the construction sector could reduce over-specification of steel (shown in [12] to account for up to 50% of steel in office blocks) and reduce yield losses (which currently account for approximately 40% of steel bought by the automotive sector [25]).

Aidt *et al.* [1] in this theme issue estimate a range of elasticity measures that explore the potential to substitute materials for other factors of production. They estimate a Hicks–Allen cross-price elasticity of substitution between materials and labour of 0.88 in the construction sector and 1.48 in the automotive sector. This implies that a 1% increase in the price of labour causes a 0.88% increase in the use of materials in the construction sector and a 1.48% increase in the use of materials in the automotive sector. Within the UK, taxes increase the cost of labour by approximately a third [26], discouraging downstream material efficiency measures that require more labour to facilitate the more efficient use of steel. Crudely extrapolating Aidt *et al.*’s substitution elasticities would then suggest that labour taxes increase material demand by 29% in the construction sector and by 48% in the automotive sector. However, this calculation is likely to over-estimate the potential for substitution between materials and labour as it is based on extrapolating results based on marginal changes in the relative prices to sizeable shifts. According to Skelton & Allwood [27], carbon prices do little to compensate for the disincentives to material efficiency caused by labour taxes: the cumulative cost of a tax set at £50/tCO_2_ on embodied emissions in key steel-using sectors does not offset the cumulative cost of labour taxes in these sectors.

#### (iii) Summary: distortions caused by government intervention

This sub-section has demonstrated that government intervention reduces incentives for downstream GhG emissions abatement activity. Unless companies are actively encouraged to make wind-fall profits by passing on the opportunity cost of freely allocated EUETS allowances, the EUETS will have little, if any, impact on incentives downstream. The UK CPS, which was introduced to impose a credible carbon price given the weak performance of the EUETS, is compensated for in such a way that energy-intensive sectors would not be expected to pass on the full cost (at the given tax rate) of the GhG emissions embodied in their production activity, thus weakening incentives downstream. Finally, the weak downstream incentives offered by carbon prices do little to compensate for the disincentives to greater material efficiency caused by labour taxes.

## 4. Distortions to the more efficient use of steel in the UK

Section 3 outlined multiple potential distortions to the incentives for downstream GhG emissions abatement. This section briefly evaluates how applicable these distortions are likely to be to the incentives for greater material efficiency in the use of steel in the UK. Section 3 began by examining possible distortions caused by the sequential nature of decision-making across supply chains, maintaining the assumption of perfect competition and exploring whether incentives hold in the transition between equilibria (§3a). The analysis showed that the sequential nature of decision-making does not, in and of itself, distort incentives (the cheapest abatement option is always chosen regardless of where along the supply chain the abatement option applies) but that viable (i.e. cheaper than the tax) but sub-optimal (i.e. not the cheapest option within the supply chain) options may be overlooked. This was shown to be a short-run problem (that would disappear over time as upstream abatement options are exhausted) but which could mean that increasing returns to abatement activity are exploited in upstream sectors before they are explored downstream. Given the low current value of carbon prices on the EUETS, the potential margin between the upstream abatement cost and the tax is small, and so this distortion is unlikely to be significant; however, should a higher carbon price (that is more in keeping with estimates of the social cost of carbon) be abruptly imposed, the potential for this type of distortion becomes greater.

Moving on to the possibility of imperfect cost pass-through explored in §3b, table 3 provides an overview of empirical estimates of the rate of cost pass-through in the steel sector. These cost pass-through rates are calculated using a range of techniques including: simulating strategic cost pass-through decisions in different theoretical models of competition (e.g. [20]), estimating a sector-specific cost function including a parameter that measures carbon price cost pass-through (e.g. [17,30]), and using econometric techniques to benchmark prices in countries where carbon taxes are applied against prices outside the jurisdiction of carbon pricing schemes (e.g. [29]).

**Table 3.** Estimates of steel sector cost pass-through (*ρ*_s_). CRC, cold rolled coil; HRC, hot rolled coil.

source	sub-sector	method	*ρ*_s_
McKinsey [28]	steel BF-BOF	expert opinion	6%
McKinsey [28]	steel EAF	expert opinion	66%
Smale *et al.*[20]	steel EAF	theoretical (Cournot)	65%
CE Delft [29]	steel HRC	empirical (equilibrium)	120%
CE Delft [29]	steel CRC	empirical (equilibrium)	110%
Vivid Economics [30]	UK steel	empirical (cost price)	75–80%
European Commission [17]	north EU HRC	empirical (cost price)	75–85%
European Commission [17]	south EU HRC	empirical (cost price)	55–100%

The results in table 3 show a large variation in estimates of cost pass-through in the steel sector, ranging from 6% to 120%. The studies span nearly a decade and focus on different sub-sectors and different geographical areas. The study by McKinsey [28] provides no evidence to substantiate its findings. Excluding this study reduces the range of estimates to 55–120%. These estimates are likely to be consequent cost pass-through estimates, i.e. they are likely to include the dampening effect of demand responses on the initial increase in prices. As explained in §3b(i), it is only if the initial cost pass-through rate is imperfect (i.e. not equal to 100%) that the unit cost of steel does not initially increase to fully reflect the emissions intensity of steel-making, and downstream incentives are distorted. Further information on the nature of competition, and the objectives of firms within the sector, is required to assess whether this is likely to be the case.

The steel industry deals in a commoditized, globally traded good. Excess capacity globally (mainly in China, but also in the EU where plants routinely operate at less than 80% capacity utilization [31]) has depressed prices, causing intense cost competition. The return on capital employed has been consistently below the weighted average cost of capital in the industry, suggesting that the industry is effectively destroying value [32]. The European Steel Association (Eurofer) [33] stresses that the need to maintain output in order to exploit economies of scale in production in this highly cost-competitive environment means that firms in the industry will opt not to initially pass on costs. To the extent that this is the case, incentives offered by the carbon tax for downstream material efficiency in the use of steel will be weakened.

Finally, §3c explored potential distortions caused by the compensation schemes that have been put in place to reduce the risk of carbon leakage. Table 2 showed the effects of two forms of compensation—free allocation of EUETS allowances and the compensation for indirect carbon costs. The compensation rate is greater for freely allocated EUETS allowances (at least 95% of GhG emissions in the UK steel sector) than the compensation rate for indirect carbon costs incurred through the UK CPS (capped at 85% and dependent on the plant’s performance relative to a benchmark); however, the carbon price is significantly higher under the UK CPS (£18/tCO_2_ compared with approximately £5/tCO_2_ under the EUETS) and firms would not be expected to pass on carbon costs for which they are compensated (under the UKCPS), whereas they would, in theory, be expected to pass on the opportunity cost of freely allocated EUETS permits. This would suggest that the the compensation for indirect carbon costs currently causes the greater distortion per unit steel.

**Figure 6.**
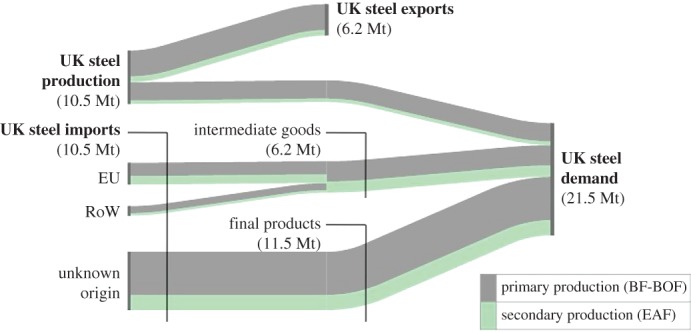
UK steel flows based on [34]. RoW, rest of the world. (Online version in colour.)

Figure 6 provides a summary of steel flows relating to the UK, including all steel that is produced in the UK (10.5 Mt) and all steel that is produced globally to meet UK demand (21.5 Mt). The UK CPS applies only to UK secondary steel-making, which supplies approximately 3% (0.7 Mt) of UK demand for steel. The EUETS covers both primary and secondary steel-making in the EU. EU production (including the UK) is known to account for 40% (8.6 Mt) of UK steel demand with a further, unknown, share of steel embodied in final goods likely to stem from the EU. The EUETS is therefore applied to a much larger mass of steel required to meet UK demand. If the opportunity cost of freely allocated allowances is not passed on (e.g. because the sector is more concerned with maintaining output as claimed by Eurofer [33]) this then becomes the principle distortion, especially if EUETS intermediate caps are tightened to bolster the carbon price. Unless firms are encouraged to make windfall profits and pass on the cost of freely obtained credits, downstream sectors do not face appropriate incentives for material efficiency in the use of steel.

## 5. Discussion

In summary, this article has sought to explore whether carbon prices are likely to offer appropriate incentives for downstream GhG emissions abatement options such as improving material efficiency in the use of steel. As discussed in the Introduction, radical change in both supply of and demand for energy is required if the highly ambitious target to limit global warming to less than 2^°^C set out in the Paris Agreement is to be met. It is therefore vital that the full gamut of abatement options are appropriately incentivized.

By putting together a simple model of sequential decision-making along supply chains, drawing on the literature on tax pass-through and on information on current carbon pricing and compensation schemes in the UK, this article has revealed that:

— The sequential nature of decision-making does not, in and of itself, distort downstream incentives for GhG abatement. If the cheapest option is to reduce carbon emissions through greater material efficiency in the use of steel then this will be incentivized, in theory, both in the post-tax equilibrium and in the transition to this equilibrium.
— As downstream sectors do not see the tax directly, but instead see the upstream abatement cost, there may be a delay in incentivizing viable (i.e. cheaper than the tax) but sub-optimal (i.e. not the cheapest within the supply chain) downstream abatement options in the transition from the pre-tax to the post-tax equilibrium. This could mean that increasing returns to abatement effort on the supply side are explored before they are explored on the demand side.
— Empirical measures of carbon price cost pass-through do not offer clear information on whether downstream incentives for material efficiency are appropriate as these measures are likely to contain an element of the demand response that is caused by the initial change in costs passed on to consumers.
— When firms operate under imperfect competition, they can restrict output to increase prices and enhance their profits. The decision on whether to pass on costs to consumers (or absorb them in profits) then becomes a strategic decision. For example, under monopoly, depending on the characteristics of the demand curve, the initial cost pass-through rate may be less than 100%, meaning that downstream sectors will not see the full cost of the embodied emissions in the materials they use.
— Under imperfect competition there is an interplay between competition policy and environmental policy: restrictions to supply under imperfect competition mean higher, pre-tax downstream material efficiency but weakened incentives for material efficiency caused by a carbon tax. In this context, the carbon tax acts partly as a tax on profits.
— Within the steel sector, imperfect initial carbon price cost pass-through rates are more likely to result from an inability of firms to unilaterally increase prices of a highly commoditized, globally traded good and from the need to focus on increasing market share to increase capacity utilization, then from imperfect competition.
— Efforts by governments to protect firms in energy-intensive sectors act to limit incentives for material efficiency downstream: freely allocated emissions permits will only incentivize downstream abatement if companies are encouraged to make windfall profits by fully passing on the opportunity cost of these permits; and compensation mechanisms that reduce the indirect cost of carbon mean that the full cost of embodied emissions is not seen by firms downstream.

Of these arguments, those raised in §3c, relating to the reality of trying to unilaterally implement a carbon price in a ‘second best’ world, are likely to be the most important for distorting incentives for downstream GhG emissions abatement through greater material efficiency in the steel sector. Governments could actively improve incentives for material efficiency by exploring three policy options:

— *Reinstate the carbon price downstream*. Neuhoff *et al.* [18] propose the ‘inclusion of consumption’ method to reinstate the carbon price in downstream sectors. Under the proposed scheme, GhG emissions allowances are issued for free (based on a benchmark plant efficiency) to energy-intensive industries. These sectors are not expected to pass on the opportunity cost associated with this free allocation. Instead a charge (dependent on the average carbon intensity of production) is levied on the consumption of carbon-intensive materials. This charge is levied on domestic and imported products alike, and takes the form of a liability that is only paid when the good is ‘released for consumption’, i.e. sold to a final consumer or exported.
— *Offer compensation for carbon leakage that is independent of embodied carbon*. If the above option (which offers incentives for greater material efficiency in the use of both domestically produced and imported materials) is not feasible, a less ambitious option would be to compensate energy-intensive industries for unilateral carbon costs through measures that do not affect the cost of carbon that they see, for example by offering corporate tax relief that is independent of energy consumption.
— *Offer strategic exemptions to other taxes that reduce incentives for material efficiency*. This has been done in Sweden, where VAT on repairs to bicycles has been reduced from 25% to 12% and where the governing Social Democratic–Green coalition has proposed measures to waive income taxes on repairs to appliances such as fridges, ovens and washing machines. These measures are explicitly aimed at reducing material consumption with a view to reducing GhG emissions.

In conclusion, carbon pricing schemes are favoured by economists because they seek to offer consistent incentives across all abatement options. This article has shown that implementing a carbon price in practice (relaxing some of the restrictive assumptions of neoclassical economics) can mean that downstream GhG emissions abatement through greater material efficiency is not duly incentivized. Tailored fiscal options have been proposed to tackle the issues raised in this paper. Suggesting these tailored fiscal instruments begs the question whether material efficiency is the most appropriate use of political effort, or whether there are other forms of abatement that are similarly unduly incentivized in a second best world that merit greater attention. Further work is required to evaluate the cost and emissions reduction potential of implementing material efficiency measures relative to other GhG emission abatement options, in order to justify these more bespoke policy instruments and to evaluate the most appropriate heuristics to allow them to be implemented.

## Appendix A

Information on how strategy costs were converted into marginal abatement costs and on how the resulting relative marginal abatement cost conditions were inferred is provided in table 4.

**Table 4.** Evaluating outcomes under sequential decision-making, where *α*_e_=*c*_e_; *α*_s_=*c*_s_/*m*_e_; *α*_c_=(*c*_c_/*a*_s,e_)/*m*_e_; *τ* represents the carbon price; *c*_*i*_ represents the cost of implementing the sector-specific abatement strategy in sector *i* (reduced carbon intensity, energy efficiency or material efficiency) in sector-specific units as defined in table 1; *m*_*i*_ represents the GhG emissions intensity per unit output in sector *i*; *a*_*i*,*j*_ represents the physical amount of output from sector *j* required to make one unit output from sector *i*; and *α*_*i*_ represents the cost of abatement in sector *i*, calculated by translating sector-specific abatement strategy costs into common £/tCO_2_ units. The sectors are denoted as follows: energy (e), steel (s) and construction (c). The final column lists all sectors that choose to abate the specific case with the sector with the lowest cost of abatement shown in bold.

case	initial inequalities	simplified inequalities	abatement?
case 1	*τ*>*c*_e_, *c*_e_⋅*m*_e_>*c*_s_, *c*_s_⋅*a*_s,e_>*c*_c_	*τ*>*α*_e_>*α*_s_>*α*_c_	e,s,**c**
case 2	*τ*>*c*_e_, *c*_e_⋅*m*_e_>*c*_s_, *c*_s_⋅*a*_s,e_<*c*_c_	*τ*>*α*_e_>*α*_s_, *α*_c_>*α*_s_	e,**s**
case 3	*τ*>*c*_e_, *c*_e_⋅*m*_e_<*c*_s_, *c*_e_⋅*m*_e_⋅*a*_s,e_>*c*_c_	*τ*>*α*_e_>*α*_c_, *α*_s_>*α*_e_	e,**c**
case 4	*τ*>*c*_e_, *c*_e_⋅*m*_e_<*c*_s_, *c*_e_⋅*m*_e_⋅*a*_s,e_<*c*_c_	*τ*>*α*_e_, *α*_s_>*α*_e_, *α*_c_>*α*_e_	**e**
case 5	*τ*<*c*_e_, *τ*⋅*m*_e_>*c*_s_, *c*_s_⋅*a*_s,e_>*c*_c_	*α*_e_>*τ*>*α*_s_>*α*_c_	s, **c**
case 6	*τ*<*c*_e_, *τ*⋅*m*_e_>*c*_s_, *c*_s_⋅*a*_s,e_<*c*_c_	*α*_e_>*τ*>*α*_s_, *α*_c_>*αs*	**s**
case 7	*τ*<*c*_e_, *τ*⋅*m*_e_<*c*_s_, *τ*⋅*m*_e_⋅*a*_s,e_>*c*_c_	*α*_e_>*τ*, *α*_s_>*τ*, *τ*>*α*_c_	**c**
case 8	*τ*<*c*_e_, *τ*⋅*m*_e_<*c*_s_, *τ*⋅*m*_e_⋅*a*_s,e_<*c*_c_	*α*_e_>*τ*, *α*_s_>*τ*, *α*_c_>*τ*	none

## References

[1] Aidt T, Jia L, Low H 2017 Are prices enough? The economics of material demand reduction. **Phil. Trans. R. Soc. A** 375, 20160370 (10.1098/rsta.2016.0370) 28461434 PMC5415648

[2] Anderson K, Le Quéré C, Mclachlan C 2014 Radical emission reductions: the role of demand reductions in accelerating full decarbonization. *Carbon Manage.* 5, 321–323. (10.1080/17583004.2014.1055080)

[3] Keohane RO, Victor DG 2011 The regime complex for climate change. *Perspect. Politics* 9, 7–23. (10.1017/S1537592710004068)

[4] Babiker MH 2005 Climate change policy, market structure, and carbon leakage. **J. Int. Econ.** 65, 421–445. (10.1016/j.jinteco.2004.01.003)

[5] Peters GP, Minx JC, Weber CL, Edenhofer O 2011 Growth in emission transfers via international trade from 1990 to 2008. *Proc. Natl Acad. Sci. USA* 108, 8903–8908. (10.1073/pnas.1006388108) 21518879 PMC3102371

[6] Grainger CA, Kolstad CD 2010 Who pays a price on carbon? **Environ. Resour. Econ.** 46, 359–376. (10.1007/s10640-010-9345-x.)

[7] Kallis G 2017 Radical dematerialization and degrowth. *Phil. Trans. R. Soc. A* 375, 20160383 (10.1098/rsta.2016.0383) 28461444

[8] Ecofys. 2015 *State and trends of carbon pricing*. Washington, DC: World Bank.

[9] Moore FC, Diaz DB 2015 Temperature impacts on economic growth warrant stringent mitigation policy. *Nat. Clim. Change* 5, 127–131. (10.1038/nclimate2481)

[10] EPA. 2016 Technical support document: Technical update of the social cost of carbon for regulatory impact analysis. United States Environmental Protection Agency, Washington, DC.

[11] Gutowski TG, Sahni S, Allwood JM, Ashby MF, Worrell E 2013 The energy required to produce materials: constraints on energy-intensity improvements, parameters of demand. *Phil. Trans. R. Soc. A* 371, 20120003 (10.1098/rsta.2012.0003) 23359744

[12] Moynihan MC, Allwood JM 2014 Utilization of structural steel in buildings. **Proc. R. Soc. A** 470, 20140170 (10.1098/rspa.2014.0170) 25104911 PMC4075790

[13] Allwood JM, Cullen JM 2011 Sustainable materials—with both eyes open. Cambridge, UK: UIT. See http://withbotheyesopen.com/.

[14] Milford RL, Pauliuk S, Allwood JM, Müller DB 2013 The roles of energy and material efficiency in meeting steel industry CO_2_ targets. **Environ. Sci. Technol.** 47, 3455–3462. (10.1021/es3031424) 23470090

[15] Varian H 1999 *Intermediate microeconomics: a modern approach*, 5th edn New York, NY: W.W. Norton.

[16] Weyl EG, Fabinger M 2013 Pass-through as an economic tool: principles of incidence under imperfect competition. *J. Political Econ.* 121, 528–583. (10.1086/670401)

[17] European Commission. 2015 *EU resource efficiency scoreboard 2014*. Brussels, Belgium: European Commision.

[18] Neuhoff K *et al.* 2016 *Inclusion of consumption of carbon intensive materials in emissions trading—an option for carbon pricing post-2020*. Cambridge, UK: Climate Strategies.

[19] Requate T 2005 *Environmental policy under imperfect competition: a survey*. See https://www.econstor.eu/bitstream/10419/22000/1/EWP-2005-12.pdf.

[20] Smale R, Hartley M, Hepburn C, Ward J, Grubb M 2006 The impact of CO_2_ emissions trading on firm profits and market prices. **Clim. Policy** 6, 31–48. (10.1080/14693062.2006.9685587)

[21] Warwick P, Ng C 2012 The ‘cost’ of climate change: how carbon emissions allowances are accounted for amongst European Union companies. *Aust. Accounting Rev.* 22, 54–67. (10.1111/j.1835-2561.2011.00158.x)

[22] BIS. 2015 *Compensation for the indirect costs of the EU emissions trading system and the carbon prices support mechanism from 2015*. London, UK: Department for Business Innovation and Skills.

[23] Griffin P, Hammond GP, Norman JB 2013 *Industrial energy use from a bottom-up perspective: developing the usable energy database (beta version)*. London, UK: Energy Research Centre.

[24] Environment Agency. 2008 *EU emissions trading scheme: further approaches to benchmarking in steel and cement sectors*. OCLC: 236121019. Bristol, UK: Environment Agency.

[25] Euro Car Body. 2016 *Car body benchmarking data*. Bad Nauheim, Germany: Euro Car Body.

[26] OECD. 2016 Taxing wages 2016. Paris, France: OECD Publishing.

[27] Skelton ACH, Allwood JM 2013 The incentives for supply chain collaboration to improve material efficiency in the use of steel: an analysis using input output techniques. *Ecol. Econ.* 89, 33–42. (10.1016/j.ecolecon.2013.01.021)

[28] McKinsey & Company. 2006 *EU ETS review: report on international competitiveness*. Brussels, Belgium: European Commission.

[29] CE Delft. 2010 *Does the energy intensive industry obtain windfall profits through the EU ETS*? Delft, The Netherlands: CE Delft

[30] Vivid Economics. 2014 *Carbon leakage prospects under phase III of the EU ETS and beyond*. London, UK: DECC.

[31] Platts P 2016 *Platts training: how the steel markets work*. Dusseldorf, Germany: Platts.

[32] Beddows R 2014 *Steel 2050: How steel transformed the world and now must transform itself*. Kingsbridge, UK: Devonian Ventures.

[33] Eurofer. 2016 *Can the steel industry pass through carbon costs without losing market shares?* Paris, France: NERA Economic Consulting

[34] EEF. 2016 *UK Steel: key statistics*. London, UK: UK Steel.

